
JOURNAL INFORMATION
==============================
NLM Title Abbreviation: Mol Genet Genomic Med
Journal ID: Mol Genet Genomic Med
NLM Journal ID: 101603758
Journal ID: MGG3

Molecular Genetics & Genomic Medicine

EISSN: 2324-9269
Publisher: Wiley

ARTICLE INFORMATION
==============================
PMCID: PMC11474623
DOI: 10.1002/mgg3.1984
Article ID: MGG31984
Article version: 1
Subjects: Issue Information, Issue Information

Issue Information

Electronic publication date: 2023 Dec 15
Publication date: 2023 Dec
Volume: 11
Issue: 12
Electronic Location ID: e1984
Copyright: © 2023 The Authors. Molecular Genetics & Genomic Medicine published by Wiley Periodicals LLC.
License: This is an open access article under the terms of the http://creativecommons.org/licenses/by/4.0/ License, which permits use, distribution and reproduction in any medium, provided the original work is properly cited.
License URL: https://creativecommons.org/licenses/by/4.0/

Figure count: 0
Table count: 0
Page count: 3

source-schema-version-number: 2.0
cover-date: December 2023
details-of-publishers-convertor: Converter:WILEY_ML3GV2_TO_JATSPMC version:6.4.9 mode:remove_FC converted:15.10.2024

==============================

MGGM

### Molecular Genetics & Genomic Medicine

Open Access

Editor-in-Chief: Suzanne Hart, PhD

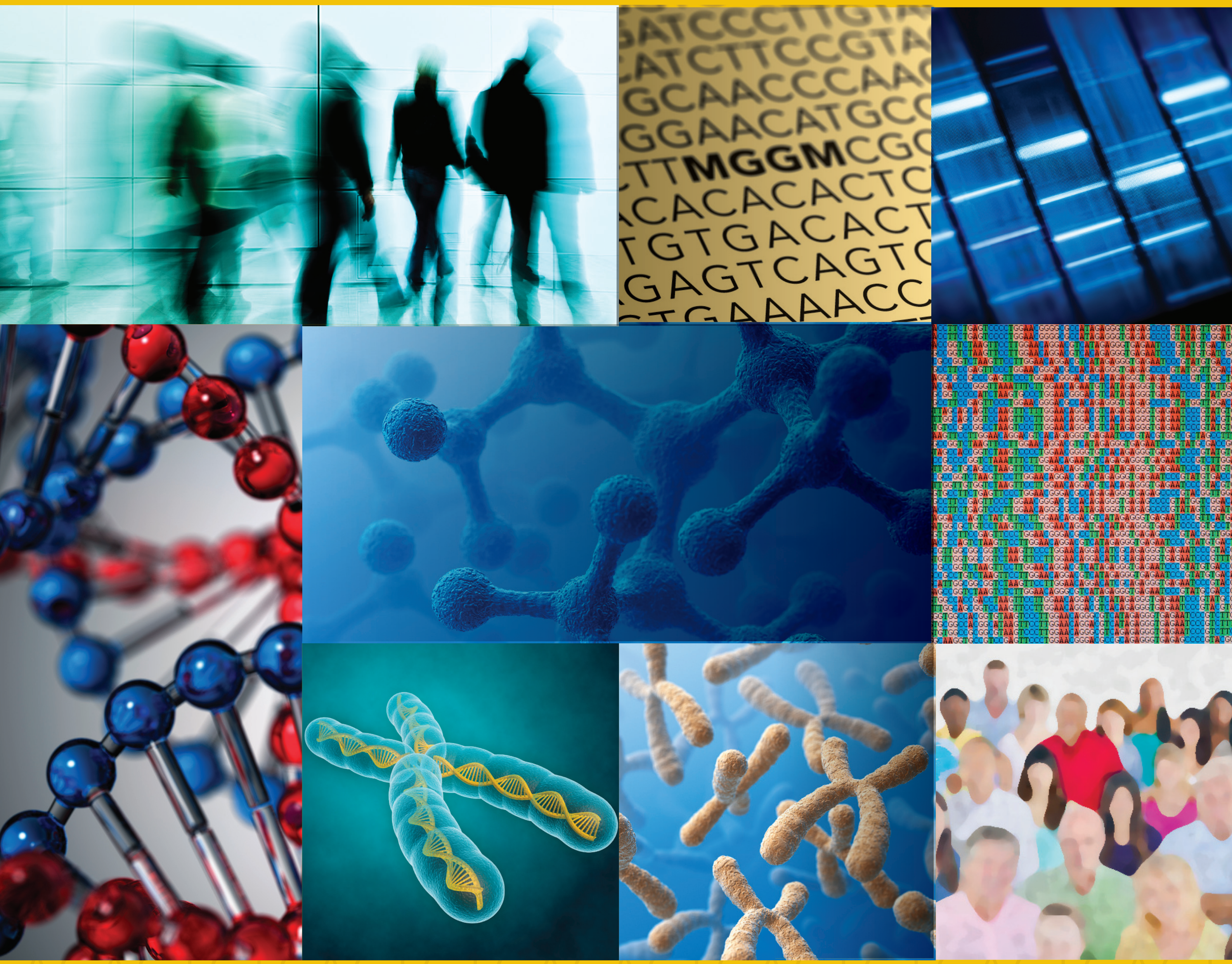

WILEY

Molecular Genetics & Genomic Medicine

Open Access

### Aims and Scope

*Molecular Genetics & Genomic Medicine* is a peer-reviewed journal for rapid dissemination of quality research related to the dynamically developing areas of human, molecular and medical genetics. The journal publishes original research articles covering findings in phenotypic, molecular, biological, and genomic aspects of genomic variation, inherited disorders and birth defects. The broad publishing spectrum of *Molecular Genetics & Genomic Medicine* includes rare and common disorders from diagnosis to treatment. Examples of appropriate articles include reports of novel disease genes, functional studies of genetic variants, in-depth genotype-phenotype studies, genomic analysis of inherited disorders, molecular diagnostic methods, medical bioinformatics, ethical, legal, and social implications (ELSI), and approaches to clinical diagnosis. *Molecular Genetics & Genomic Medicine* provides a scientific home for next generation sequencing studies of rare and common disorders, which will make research in this fascinating area easily and rapidly accessible to the scientific community. This will serve as the basis for translating next generation sequencing studies into individualized diagnostics and therapeutics, for day-to-day medical care.

*Molecular Genetics & Genomic Medicine* publishes original research articles, reviews, and research methods papers, along with invited editorials and commentaries. Original research papers must report well-conducted research with conclusions supported by the data presented.

*Molecular Genetics & Genomic Medicine* is a Wiley Open Access journal, one of a series of peer reviewed titles publishing quality research with speed and efficiency. For further information visit the Wiley Open Access website.

### Open Access and Copyright

All articles published by *Molecular Genetics & Genomic Medicine* are fully open access: immediately freely available to read, download and share. All *Molecular Genetics & Genomic Medicine* articles are published under the terms of the Creative Commons Attribution License which permits use, distribution and reproduction in any medium, provided the original work is properly cited.

Copyright on any research article in a journal published by *Molecular Genetics & Genomic Medicine* is retained by the author(s). Authors grant Wiley a license to publish the article and identify itself as the original publisher. Authors also grant any third party the right to use the article freely as long as its integrity is maintained and its original authors, citation details and publisher are identified. Further information about open access license and copyright can be found here - https://authorservices.wiley.com/author-resources/Journal-Authors/open-access/hybrid-open-access.html

### Purchasing Print Reprints

As this is an open access journal, you have free, unlimited access to your article online. However, if you wish to obtain printed reprints, these may be ordered online: https://caesar.sheridan.com/reprints/redir.php?pub=10089&acro=MGG3

### Disclaimer

The Publisher, Molecular Genetics & Genomic Medicine and Editors cannot be held responsible for errors or any consequences arising from the use of information contained in this journal; the views and opinions expressed do not necessarily reflect those of the Publisher, Molecular Genetics & Genomic Medicine or Editors, neither does the publication of advertisements constitute any endorsement by the Publisher, Molecular Genetics & Genomic Medicine or Editors of the products advertised.

For submission instructions, subscription and all other information visit:
https://onlinelibrary.wiley.com/page/journal/23249269/homepage/publishing_with_molecular_genetics___genomic_medicine.htm
View this journal online at wileyonlinelibrary.com/journal/MGGM

Molecular Genetics & Genomic Medicine

Open Access

**Editor-in-Chief**

Suzanne Hart, Ph.D.
*National Human Genome Research Institute, NIH, Bethesda, Maryland, USA**

**Associate Editors**

Lisa McReynolds, M.D., Ph.D.
*National Cancer Institute, Maryland, USA**

Sho Yano, M.D., Ph.D.
*National Human Genome Research Institute, NIH, Bethesda, Maryland, USA**

Yingze Zhang, Ph.D.
*University of Pittsburgh, Pennsylvania, USA*

Changrui Xiao, M.D.
*University of California, California, USA*

April Adams, M.D., M.S.
*Baylor College of Medicine, Texas, USA*

**Deputy Associate Editors**

Astrid Rodriguez-Acevedo, Ph.D.
*University of Queensland Diamantina Institute, Brisbane, Australia*

*These editors serve in their own capacity. The views expressed are their own and do not necessarily represent the views of the National Institutes of Health or the United States Government.

**Editorial Board Members**

Yair Anikster
*Sheba Medical Center, Israel*

Mauricio Arcos-Burgos
*Universidad del Rosario, Columbia*

Nurten A. Akarsu
*Hacetepe University, Turkey*

Michael Bamshad
*University of Washington School of Medicine, USA*

María Leonor Bustamente Calderón
*University of Chile, Chile*

Véronique David
*University of Rennes, France*

Mauricio De Castro
*Air Force Medical Genetics Center, USA*

Michael J. Gambello
*Emory University, USA*

Andrea Gropman
*Children's National Medical Center, USA*

Robert Kleta
*University College of London, UK*

Stanislav Kmoch
*Charles University in Prague, Czech Republic*

Maria Rita Passos-Bueno
*University of Sao Paulo, Brazil*

Bruno Reversade
*A*STAR, Singapore*

Joris Veltman
*Radboud University Nijmegen Medical Centre, The Netherlands*
